# Association of genetic ancestry with pre-eclampsia in multi-ethnic cohorts of pregnant women

**DOI:** 10.1016/j.preghy.2024.101162

**Published:** 2024-12

**Authors:** Frances Conti-Ramsden, Antonio de Marvao, Carolyn Gill, Lucy C. Chappell, Jenny Myers, Dragana Vuckovic, Abbas Dehghan, Pirro G. Hysi

**Affiliations:** a Department of Women and Children’s Health, School of Life Course & Population Sciences, King’s College London, UK; b British Heart Foundation Centre of Research Excellence, School of Cardiovascular and Metabolic Medicine and Sciences, King’s College London, UK; c Medical Research Council Laboratory of Medical Sciences, Imperial College London, UK; d Division of Developmental Biology and Medicine, University of Manchester, UK; e Department of Epidemiology and Biostatistics, Imperial College London, UK; f MRC Centre for Environment and Health, School of Public Health, Imperial College London, UK; g UK Dementia Research Institute, Imperial College London, UK; h Section of Ophthalmology, School of Life Course & Population Sciences, King’s College London, UK; i Department of Twin Research & Genetic Epidemiology, King’s College London, UK

**Keywords:** Pregnancy, Pre-eclampsia, Ethnicity, Ancestry

## Abstract

**Objectives:**

Maternal self-reported ethnicity is recognised as a risk factor for pre-eclampsia in clinical screening tools and models. This study investigated whether ethnicity is acting as a proxy for genetic variants in this context.

**Study design:**

A total of 436 women from multi-ethnic backgrounds recruited to two UK observational pregnancy hypertension cohort studies were genotyped. Genetically-computed individual ancestry estimates were calculated for each individual through comparison to the multi-ethnic 1000 Genomes reference panel genotypes. Regression models for pre-eclampsia using clinical risk factors including self-reported ethnicity with and without ancestry estimates were built and compared using Likelihood Ratio Tests (LRT).

**Main outcome measures:**

Pre-eclampsia (early- and late-onset).

**Results:**

In these multi-ethnic cohorts (mean age 34.9 years; 41.3 % White, 34.2 % Black, 13.1 % Asian ethnic backgrounds; 82.6 % chronic hypertension), discrepancies between self-reported ethnicity and genetically-computed individual ancestry estimates were present in all ethnic groups, particularly minority groups. Genetically-computed pan-African ancestry percentage was associated with early-onset (< 34 weeks) pre-eclampsia in adjusted models (aOR 100 % vs 0 % African ancestry: 3.81, 95 % CI 1.04–14.14, p-value 0.044) independently of self-reported ethnicity and established clinical risk factors. Addition of genetically-computed African ancestry to a clinical risk factor model including self-reported ethnicity, improved model fit (Likelihood ratio test p-value 0.023).

**Conclusions:**

Self-reported maternal ethnicity is an imperfect proxy for genetically-computed individual ancestry estimates, particularly in ethnic minority groups. Genetically-computed African ancestry percentage was associated with early-onset pre-eclampsia independently of self-reported maternal ethnicity. Well-powered studies in multi-ethnic cohorts are required to delineate the genetic contribution to pre-eclampsia.

## Highlights

• Maternal ethnicity is an imperfect proxy for genetic ancestry in multi-ethnic populations of UK pregnant women.
• Genetic ancestry may be a better predictor of early-onset pre-eclampsia than maternal ethnicity.
• Larger genetic studies in multi-ethnic cohorts are needed to determine genomic contribution to pre-eclampsia risk.

## 1 Introduction

Pre-eclampsia affects 3–5 % of pregnancies worldwide and causes substantial maternal and infant mortality and morbidity [1], [2], [3]. Compared to a general antenatal population, the risk of pre-eclampsia is approximately five times higher in women with pre-existing chronic hypertension (17–25 %) [4], [5]. Aspirin is currently the sole preventative drug [6], and is most effective when initiated prior to 16 weeks’ gestation [7]. Early prediction of pre-eclampsia is thus required to optimise risk stratification in maternity care and guide aspirin prophylaxis [8].

The incidence and timing of onset of pre-eclampsia varies significantly across maternal ethnic groups in epidemiological studies, typically using self-reported ethnicity [9], [10]. Women of non-Hispanic Black ethnic backgrounds are at the highest risk even after adjustment for potential confounding factors [10], [11]. Black maternal ethnic background (or related categories such as African American) is included as an independent risk factor in several international guidelines recommending aspirin prophylaxis [4], and is also included in the Fetal Medicine Foundation (FMF) pre-eclampsia prediction algorithm [12], [13], [14], which combines established maternal clinical risk factors (such as maternal age, chronic hypertension, diabetes, chronic kidney disease and in vitro fertilisation) with ultrasound and biochemical parameters [8], [15].

However, the use of ethnicity in clinical risk assessments is controversial [16]. Ethnic categories represent socio-political constructs with complex links to ancestry [17]. They may be proxies for social factors rather than biological determinants of health [16], [18], raising concerns about using self-reported ethnicity in clinical algorithms, where their use may reify the notion of biological differences between ethnic groups.

Genetically-computed individual ancestry estimates, a feature of an individual’s inherited genome, can be used as an alternative or alongside self-reported ethnicity to disentangle social, environmental and genetic factors in disease [19], [20]. Individuals may not be fully aware of their own ancestry and as modern populations become increasingly genetically admixed, self-allocation of a single ethnic group is likely to be an increasingly unrepresentative proxy for population ancestry [19].

This study investigates the relationship between self-reported maternal ethnicity and genetically-computed individual ancestry estimates (referred to as genetic ancestry in further text) in contemporaneous, multi-ethnic UK antenatal populations and investigate whether genetic ancestry has clinical applicability by evaluating its association with the development of pre-eclampsia and adverse pregnancy outcomes.

## 2 Methods

The data that support the findings of this study are available from the corresponding author upon reasonable request.

### 2.1 Cohort description

Samples and clinical data were obtained from individuals recruited to two observational hypertensive disorder of pregnancy (HDP) cohort studies who had given consent for genetic studies (Research Ethics Committee approval PEACHES: 11/LO/1776 (2013), MAVIS – Tommy’s Tissue Bank: 15/NW/0829 (2015)). The PEACHES cohort recruited pregnant women from a South London population between 2013–2021 [21]. The MAVIS cohort recruited pregnant women from a Central Manchester population between 2017–2019. Women recruited with chronic (pre-existing) hypertension and women normotensive at study entry (no chronic hypertension group) with and without risk factors for hypertensive disorders of pregnancy (HDP) (history of HDP in a previous pregnancy, high-risk medical condition or in vitro fertilisation) with singleton pregnancies were included in this study. In both studies, pregnancy and outcome data were recorded in dedicated electronic databases prospectively during pregnancy and following delivery by case note review.

### 2.2 Study variable definitions

Recorded maternal ethnic groups were harmonised into detailed ethnic groupings, collapsed ethnic category groupings in line with UK Office of National Statistics (ONS) groups [22] and ethnic categories used in the FMF pre-eclampsia risk prediction model (Supplementary Table 1). Participant postcodes were linked to lower super output area (LSOA) Index of multiple deprivation scores using English indices of deprivation 2015 or 2019 as appropriate. [23], [24] Where women had no fixed abode (n = 2), the most deprived category was assigned.

Final outcome diagnosis, e.g. superimposed pre-eclampsia, was assigned following a review of clinical notes. Where there was ambiguity, the research database and relevant hospital and laboratory records were reviewed by a clinician and a final outcome was assigned according to the 2018 International Society for Study of Hypertension in Pregnancy definitions of hypertensive disorders of pregnancy [3]. Early- and late-onset pre-eclampsia were defined as pre-eclampsia diagnosis with birth at < 34 weeks’ or ≥ 34 weeks’ gestation respectively. Birthweight centiles were calculated using the INTERGROWTH calculator (http://intergrowth21.ndog.ox.ac.uk/en/Upload/Upload).

Details of genotyping, genomic quality control and ancestry calculation are available in the supplementary methods.

### 2.3 Statistical analysis

The relationship between maternal self-reported ethnicity and genetically-computed individual ancestry estimates in the whole study cohort was assessed through visual inspection of ancestry proportion plots and Sankey plots. Genetically-computed individual ancestry estimates are summarised as proportions summing to 1 per individual, obliging strong correlation between ancestry proportions. There was a high negative correlation between African and European superpopulation ancestry, as well as the Northern/Western European population ancestry (CEU) and most other non-European genetic ancestries, particularly West African Yoruban ancestry (YRI) in this cohort (Supplementary Fig. 2). Therefore, to prevent collinearity, genetic ancestry estimates were inputted individually into models and European ancestries were not included. Furthermore, due to the very low prevalence of East Asian and Admixed American genetic ancestries in this cohort (n = 10 and n = 7 individuals with >10 % East Asian and Admixed American genetic ancestries respectively) these individual ancestries were not tested in models due to high likelihood of spurious results. In view of the high genetic diversity within African populations [25], individual African population ancestries present at a prevalence of >3 % across the whole study cohort were inputted individually into models in place of African superpopulation ancestry proportion in sensitivity analyses. These were West African Yoruban (YRI) and East African Luhya (LWK) population ancestries (Supplementary Fig. 3).

To further assess whether individual genetic ancestry estimates have the potential to improve existing pre-eclampsia risk prediction algorithms two multivariable regression models were formulated. In model A all established risk factors for pre-eclampsia as per international clinical guidelines [4] and the Fetal Medicine Foundation pre-eclampsia algorithm [13] available in the datasets were regressed onto the outcome of pre-eclampsia (early or late onset). These were: maternal age, ethnicity, parity, history of pre-eclampsia in a previous pregnancy, body mass index, smoking status, chronic kidney disease, Type I diabetes, Type II diabetes, IVF pregnancy and mean arterial pressure. For model B, in addition to all the variables included in model A, continuous, genetic ancestry proportions were included in the model. An Analysis of variance (ANOVA) likelihood ratio test was used to determine whether the addition of genetic ancestry estimates significantly improved model fit. Improvement in model explained variance was also assessed by computation of pseudo-R squared as defined by Veall and Zimmermann [26].

For the multivariable models, ethnic categories were defined as per Fetal Medicine Foundation groupings (Black, East Asian, Mixed, South Asian, White, Other/Unknown). [4] Due to very small numbers of women in the East Asian ethnic group (n = 4), these individuals were grouped with the Other/Unknown group to prevent spurious results.

The Index of multiple deprivation, which is not currently included in the Fetal Medicine Foundation pre-eclampsia risk prediction algorithm or international guidelines but is a potential confounding factor in the association between ethnicity and pre-eclampsia, was added to both models in sensitivity analyses.

Logistic regression model assumptions were checked by assessing the distribution of residuals. As the proportion of individuals with missing data was low (< 5 %), complete case analysis was undertaken for all models. Analysis was completed using the base package of the R statistical environment, version 4.2.1 (2022–06-23), [27] admixture plots were generated using the R pophelper package [28], sankey plots were generated using the R package networkD3 [29].

## 3 Results

Of 467 women meeting study inclusion criteria, genotype data passed genotyping quality control (QC) for 436 (93.4 %) and were used for ancestry analysis. After accounting for miscarriage, termination of pregnancy and loss to follow-up, 421 of 436 individuals (96.6 %) had complete pregnancy outcome data. Baseline characteristics and maternal and infant outcomes stratified by recruitment status are shown in Table 1. Most (347) women had chronic hypertension. The incidence of pre-eclampsia was 20.7 % in women recruited with chronic hypertension, and 6.8 % in women normotensive at entry to pregnancy, reflecting a high proportion of individuals with other conditions with higher risk of pre-eclampsia in this group (women with chronic kidney disease, diabetes mellitus and IVF pregnancy).

**Table 1:** Baseline characteristics, maternal and neonatal outcomes stratified by recruitment status. Maternal and infant outcomes were available in 421/436 (96.6 %) of individuals. Categorical variables are summarised as counts and proportions. Continuous variables are summarised as mean (standard deviation) or median [interquartile range]. *IVF =* in vitro *fertilisation, IMD = index of multiple deprivation, CS = caesarean section.*

	All	Chronic hypertension	No chronic hypertension
**Baseline maternal characteristics (n = 436)**			
n	436	360	76
Age (years)	34.9 (5.3)	35.3 (5.3)	32.8 (4.9)
Body mass index (kg/m^2^)	28.0 [23.8, 33.0]	28.7 [24.4, 34.0]	25.0 [22.0, 29.0]
Primiparous	146 (33.5)	121 (33.6)	25 (32.9)
*Missing*	1 (0.2)	1 (0.3)	0 (0.0)
Ethnicity			
*Asian*	57 (13.1)	35 (9.7)	22 (28.9)
*East Asian*	4 (0.9)	4 (1.1)	0 (0.0)
*South Asian*	43 (9.9)	25 (6.9)	18 (23.7)
*Other Asian*	8 (1.8)	6 (1.4)	4 (4.2)
*Black*	149 (34.2)	134 (37.2)	15 (19.7)
*African*	119 (27.3)	106 (29.4)	13 (17.1)
*Caribbean*	30 (6.9)	28 (7.8)	2 (2.6)
*Mixed*	17 (3.9)	13 (3.6)	4 (5.3)
*Other*	25 (5.7)	21 (5.8)	4 (5.3)
*White*	180 (41.3)	151 (41.9)	29 (38.2)
*Unknown*	8 (1.8)	6 (1.7)	2 (2.6)
Chronic kidney disease	43 (9.9)	31 (8.6)	12 (15.8)
Type 1 Diabetes mellitus	11 (2.5)	10 (2.8)	1 (1.3)
IVF pregnancy	41 (9.4)	37 (10.3)	4 (5.3)
*Missing*	4 (0.9)	3 (0.8)	1 (1.3)
IMD quintile			
*1 (lowest)*	160 (36.7)	137 (38.1)	26 (34.2)
*2*	134 (30.7)	110 (30.6)	24 (31.6)
*3*	59 (13.5)	49 (13.6)	10 (13.2)
*4*	41 (9.4)	35 (9.7)	6 (7.9)
*5 (highest)*	28 (6.4)	23 (6.4)	5 (6.6)
*Missing*	14 (3.2)	6 (1.7)	5 (6.6)
PEACHES cohort	239 (54.8)	203 (56.4)	37 (48.7)

**Maternal and neonatal outcomes (n = 421)**			
n	421	347	74
Gestation at delivery (weeks)	38.4 [37.3, 39.3]	38.3 [37.1, 39.0]	39.2 [37.9, 40.3]
Onset of labour			
*Induction*	198 (47.0)	170 (49.0)	28 (37.8)
*Pre-labour CS*	152 (36.1)	130 (37.5)	22 (29.7)
*Spontaneous*	61 (14.5)	39 (11.2)	22 (29.7)
*Missing*	10 (2.4)	8 (2.3)	2 (2.7)
Mode of delivery			
*Elective CS*	178 (42.3)	154 (44.4)	24 (32.4)
*Emergency CS*	53 (12.6)	47 (13.5)	6 (8.1)
*Vaginal*	156 (37.1)	121 (34.9)	35 (47.3)
*Vaginal assisted*	34 (8.1)	25 (7.2)	9 (12.2)
Pre-eclampsia	77 (18.3)	72 (20.7)	5 (6.8)
*Early-onset*	29 (6.9)	27 (7.8)	2 (2.7)
*Late-onset*	48 (11.4)	45 (13.0)	3 (4.1)
Composite adverse maternal outcome*			
*Missing data*	191 (43.8) 8 (1.8)	166 (46.1) 7 (1.9)	25 (32.9) 0 (0.0)
Stillbirth	6 (1.4)	6 (1.7)	0 (0.0)
Infant birthweight, g	3040 [2625, 3430]	3020 [2600, 3406]	3256 [2783, 3569]
Infant birthweight <10th centile	53 (12.6)	46 (13.3)	7 (9.5)
*Missing*	2 (0.5)	2 (0.6)	0 (0.0)
Infant admission to Neonatal Unit	70 (16.6)	64 (18.4)	6 (8.1)

* Composite of adverse maternal outcomes was defined as one or more of the following: eclampsia, placental abruption, liver haematoma or rupture, postpartum haemorrhage (estimated blood loss >500 mL if vaginal delivery, > 1000 mL if caesarean section), hepatic dysfunction (alanine transaminase >40 IU/L), thrombocytopaenia (Platelet count < 150 × 10^9^/L) or acute kidney injury (diagnosis in clinical notes or creatinine >90 μmol/L in absence of chronic kidney disease).

### 3.1 Comparison of maternal ethnicity and genetic ancestry

**Fig. 1:**
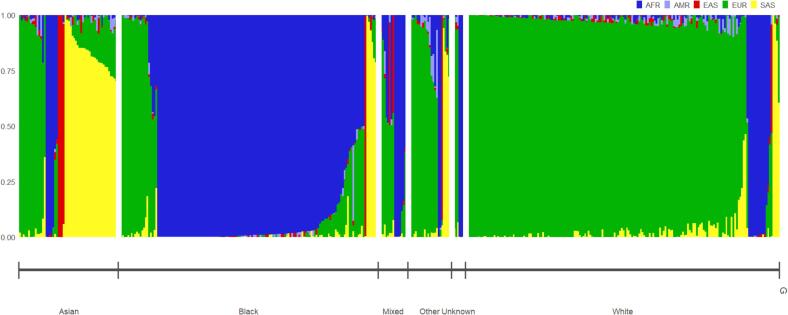
Genetically-estimated individual ancestry proportions (AFR: African, AMR: Admixed American, EAS: East Asian, EUR: European, SAS: south Asian superpopulations, calculated using 1000 Genomes as reference data) in study cohort (n = 436). Each individual is denoted by a single vertical stacked bar, with individual population ancestry percentages (summing to 1) denoted by bar colouring. Individuals are grouped by self-reported ethnic group.

**Fig. 2:**
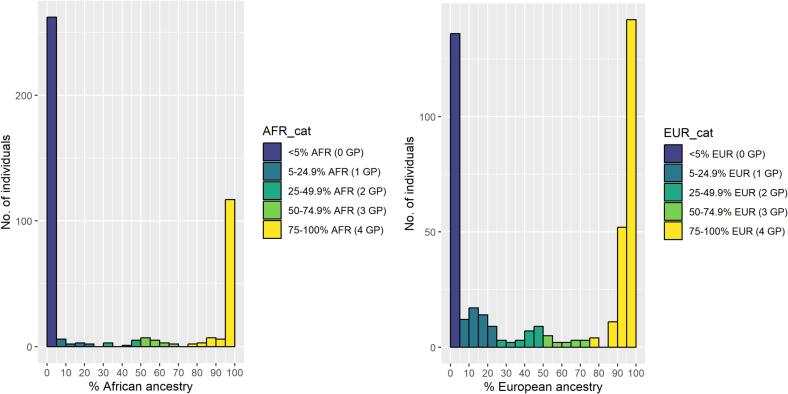
Histograms of percentage African (A) and European (B) ancestry in whole study cohort (n = 436), with frequency bars coloured by number of ‘equivalent grandparents’. *AFR = African, EUR = European, GP = grandparent(s).*

**Fig. 3:**
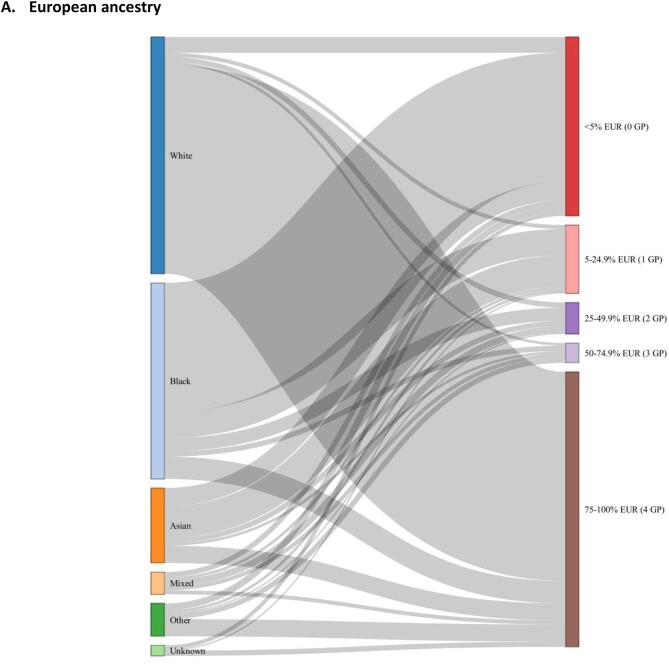

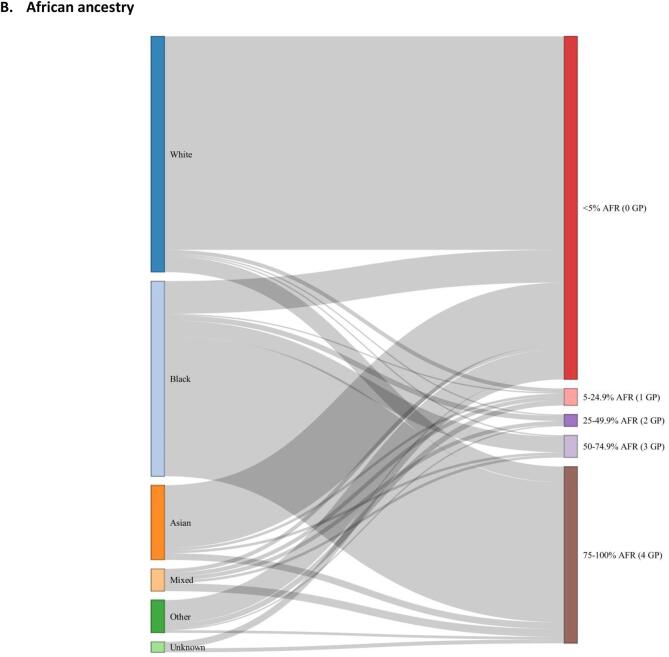

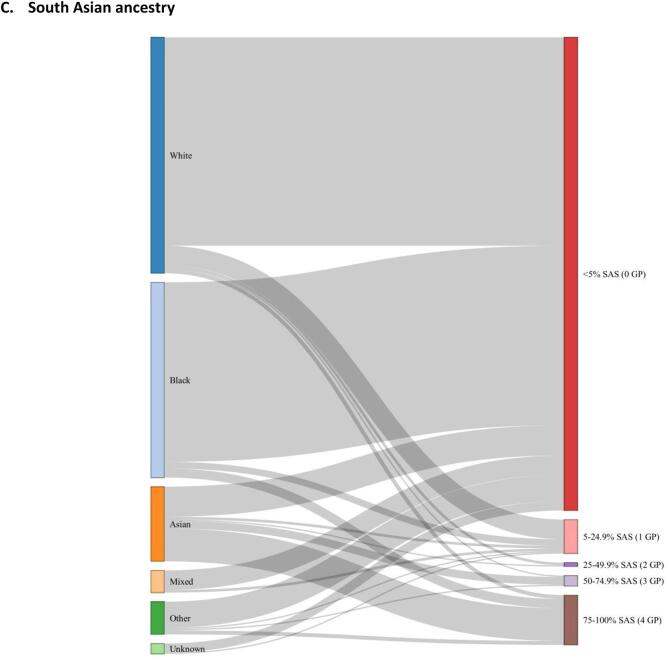
Sankey plots linking self-reported ethnic group (left hand panel) to genetically-estimated ancestry percentages: A) European (EUR), B) African (AFR), C) South Asian (SAS). Genetically-estimated ancestry percentages are grouped by number of ‘equivalent grandparents’ (GP).

The distribution of genetic ancestry estimates (European, African, South Asian, East Asian and Admixed American superpopulations) for all individuals (n = 436) are shown in Fig. 1. European and African genetically-computed ancestries were the most common ancestries in the cohort (Fig. 2**)**, and they followed a bimodal distribution. Self-reported ethnic group generally aligned with percentage European, African and South Asian genetic ancestry albeit imperfectly (Fig. 3, Supplementary Table 3). The disparity between genetic ancestry and self-reported ethnicity was present at various degrees across all ethnic groups (Fig. 1**,** Fig. 3), but was most notable in ethnic minority groups. For example, almost 20 % of individuals who reported Black ethnic backgrounds had less than 50 % African genetic ancestry, and over 25 % of individuals reporting Asian ethnic backgrounds had greater than 50 % European genetic ancestry (Fig. 3**,** Supplementary Table 3).

East Asian and Native American ancestries were rare in the study cohort (n = 10 and n = 7 individuals with >10 % East Asian and Native American genetic ancestries respectively), for which reason these ancestries were excluded from regression models.

### 3.2 Baseline model for pre-eclampsia including self-reported maternal ethnicity

A multivariable model was built for early- and late-onset pre-eclampsia using established clinical risk factors (Table 2, Table 3) from the 409 women with complete outcome and covariate data. Self-reported maternal ethnicity was not significantly associated with early- or late-onset pre-eclampsia, though direction and magnitude of effects for the Black maternal ethnic group aligned with prior studies (Table 2**,** Table 3) [30], [9].

**Table 2:** Multivariable (aOR) logistic regression analysis of early-onset pre-eclampsia outcomes using established clinical risk factors alone (model A) and clinical risk factors and genetically-estimated ancestry percentages (B). *All clinical risk factors listed in the table (Ethnicity, previous pre-eclampsia etc) were included in models A and B with results displayed as adjusted odds ratios.*

	Model A: Clinical risk factors			Model B: Clinical risk factors + genetically-estimated ancestry percentages		
	aOR	95 % CI	p-value	aOR	95 % CI	p-value
***Early onset pre-eclampsia (delivery < 34 weeks)***	**N = 409**			**N = 409**		
Genetic ancestry (1 % increments)	Not included	Not included	Not included			
% African ancestry	−	−	−	**1.01**	**1.00**–**1.03**	**0.044**
% South Asian ancestry	−	−	−	1.00	0.97–1.02	0.985
Ethnicity (reference: White)	−	−	−	−	−	−
*Black*	2.00	0.73–5.79	0.185	0.82	0.21–3.22	0.779
*South Asian*	1.00	0.14–4.50	0.996	0.97	0.1–6.15	0.972
*Mixed*	2.28	0.28–11.88	0.370	1.27	0.14–7.46	0.807
*Other/Unknown*	0.81	0.12–3.48	0.793	0.69	0.1–3.13	0.663
Previous pre-eclampsia	**3.62**	**1.28**–**10.89**	**0.017**	**4.02**	**1.4**–**12.32**	**0.011**
Chronic hypertension	1.70	0.39–11.91	0.521	1.44	0.33–10.16	0.662
Mean Arterial Pressure (in cmHg)	**1.50**	**1.01**–**2.29**	**0.048**	**1.57**	**1.05**–**2.41**	**0.031**
Age (years)	0.99	0.91–1.08	0.824	0.99	0.91–1.08	0.864
Multiparity	0.55	0.18–1.67	0.292	0.55	0.18–1.65	0.280
BMI (kg/m^2^)	0.99	0.92–1.06	0.790	0.99	0.92–1.06	0.753
Smoker	1.15	0.06–6.86	0.898	1.3	0.07–8.11	0.814
Chronic kidney disease	**3.60**	**0.99**–**11.57**	**0.038**	**3.71**	**0.99**–**12.32**	**0.038**
Pre-existing Diabetes	1.65	0.18–8.93	0.602	1.72	0.19–9.97	0.582
In Vitro Fertilisation	1.86	0.35–7.66	0.417	2.05	0.38–8.76	0.360

**Table 3:** Multivariable (aOR) logistic regression analysis of late-onset pre-eclampsia outcomes using established clinical risk factors alone (model A) and clinical risk factors and genetically-estimated ancestry percentages (B). *All clinical risk factors listed in the table (Ethnicity, previous pre-eclampsia etc) were included in models A and B with results displayed as adjusted odds ratios.*

	Model A: Clinical risk factors			Model B: Clinical risk factors + genetically-estimated ancestry percentages		
	aOR	95 % CI	p-value	aOR	95 % CI	p-value
***Late onset pre-eclampsia (delivery ≥ 34 weeks)***	**N = 409**			**N = 409**		
Genetic ancestry (1 % increments)	Not included	Not included	Not included			
% African ancestry				0.99	0.98–1.00	0.291
% South Asian ancestry				1.01	0.99–1.02	0.322
Ethnicity (ref White)	−	−	−	−	−	−
*Black*	2.17	0.97–5.04	0.064	**3.13**	**1.08**–**9.26**	**0.037**
*South Asian*	0.27	0.01–1.47	0.218	0.18	0.01–1.28	0.141
*Mixed*	2.85	0.64–10.73	0.138	3.74	0.79–15.12	0.074
*Other/Unknown*	2.17	0.74–5.93	0.139	2.07	0.69–5.79	0.176
Previous pre-eclampsia	**4.32**	**1.85**–**10.69**	**0.001**	**4.09**	**1.74**–**10.21**	**0.002**
Chronic hypertension	3.46	1.05–15.79	0.064	**3.88**	**1.16**–**18.01**	**0.046**
Mean Arterial Pressure (mmHg)	0.99	0.96–1.02	0.502	0.99	0.96–1.02	0.455
Age (years)	1.04	0.97–1.11	0.292	1.04	0.97–1.12	0.260
Multiparity	**0.31**	**0.12**–**0.76**	**0.012**	**0.30**	**0.12**–**0.73**	**0.010**
BMI (kg/m^2^)	1.02	0.96–1.07	0.575	1.01	0.96–1.07	0.625
Smoker	0.65	0.03–3.74	0.690	0.65	0.03–3.81	0.697
Chronic kidney disease	1.70	0.51–4.87	0.352	1.7	0.51–4.86	0.349
Pre-existing Diabetes	**5.84**	**1.33**–**25.64**	**0.017**	**6.00**	**1.39**–**26.06**	**0.014**
In Vitro Fertilisation	1.08	0.30–3.35	0.904	1.03	0.28–3.25	0.957

### 3.3 Association of genetically-computed pan-African ancestry with early-onset pre-eclampsia

After adding African and South Asian genetic ancestries to the baseline clinical risk factor model (Table 2, Table 3**,** models B), each percentage increase in African ancestry was associated with higher risk of early-onset pre-eclampsia independent of all other established clinical risk factors including self-reported ethnicity, BMI and baseline mean arterial pressure (ancestry-adjusted Odds Ratio, aOR = 1.01, 95 % CI 1.00–1.03, p = 0.044 per 1 % increment, for a full list of model covariates see Table 2) (Table 2, model B). Comparing 100 % vs 0 % African ancestry rather than modelling a 1 % increase in African ancestry yielded an aOR 3.81, 95 % CI 1.04–14.14, p-value 0.044 in the same model adjusted for established clinical risk factors as displayed in Table 2. Using categorised measures of African ancestry in the model yielded comparable results (aOR early-onset pre-eclampsia 75–100 % African ancestry (4 ‘equivalent grandparents’) vs < 5 % African ancestry: aOR 3.50, 95 % CI 0.96–13.00, p-value 0.058). Neither African nor South Asian ancestry was associated with late-onset pre-eclampsia (Table 3).

### 3.4 Addition of genetically-computed pan-African ancestry to early-onset pre-eclampsia model

Addition of African genetic ancestry percentage to the baseline early-onset pre-eclampsia model (model A, including self-reported ethnicity) improved model fit (likelihood ratio test (LRT) clinical risk factor model A + African genetic ancestry, p = 0.032) and increased model explained variance (pseudo R^2^) from 18.13 % to 22.50 % [26].

### 3.5 Genetically-computed West African ancestry and early-onset pre-eclampsia

We further explored the association between African genetic ancestries and early-onset pre-eclampsia by considering specific African populations from the 1000 Genomes population: YRI – Yoruba in Ibadan Nigeria (West African) and LWK − Luhya in Webuye Kenya (East African) **(**Supplementary Fig. 3**)**. We found that Yoruban (West African) genetic ancestry was significantly associated with early-onset pre-eclampsia independently of covariates including self-reported ethnicity (aOR per 1 % increment Yoruban ancestry: 1.02, 95 % CI 1.00–1.03, p-value 0.037), with a higher odds ratio than that estimated for pan-African genetic ancestry. Addition of Yoruban ancestry percentage to the baseline early-onset pre-eclampsia model (model A) also brought about an improvement in model fit (LRT clinical risk factor model A + Yoruban ancestry, p = 0.023) and increase in model pseudo R^2^ from 18.13 % to 22.32 %.

There was no significant association between Luhya (East African) genetic ancestry and early-onset pre-eclampsia (aOR per 1 % increment Luhya ancestry: 0.99, 95 % CI 0.95–1.02, p-value 0.567); however, this analysis was underpowered as demonstrated by the wide confidence intervals.

### 3.6 Early-onset pre-eclampsia model sensitivity analyses

In a sensitivity analysis, index of multiple deprivation (a proxy for socio-economic status) was included as a model covariate due to potential for confounding between self-reported ethnicity, ancestry and socio-economic status. Addition to the model (total number analysed n = 404) did not substantially alter results, with a significant association remaining between pan-African genetic ancestry and early-onset pre-eclampsia (aOR per 1 % increment in pan-African ancestry: 1.01, 95 % CI 1.00–1.03, p = 0.035). In a model in which self-defined ethnicity was excluded, pan-African genetic ancestry also was significantly associated with early-onset pre-eclampsia (aOR per 1 % increment in pan-African ancestry: 1.01, 95 % CI 1.00–1.02, p-value 0.009), and improved model fit when added to the model (likelihood ratio test p-value: 0.009).

## 4 Discussion

We have demonstrated that in urban UK populations, self-reported ethnicity does not reliably align with individual genetically-computed ancestry, particularly in ethnic minority groups. We further showed that African genetic ancestry, particularly West African Yoruban population genetic ancestry, is strongly associated with early-onset pre-eclampsia independently of self-reported ethnicity and established clinical risk factors including BMI and baseline BP in a high-risk cohort. Due to sample size limitations, the findings are primarily relevant to Yoruban population ancestry, which is the predominant ethnic origin of British individuals of African ancestry. As addition of either pan-African genetic ancestry or Yoruban genetic ancestry to a multivariable clinical risk factor model improved model fit and explained variance, pre-eclampsia risk prediction could be improved by the addition of genetic data.

Eurocentric bias is a recognised problem in contemporary genomics [31], exemplified by the recently published Genome-Wide Association Study (GWAS) and *meta*-analysis for pre-eclampsia which included just 20 cases in women of Black ethnic backgrounds out of a total of 20,064 cases [32]. Ours is the largest genetic study of pre-eclampsia in a truly multi-ethnic cohort representative of contemporary, urban UK maternity populations. Whilst the sample size was insufficient for GWAS, use of a genetic ancestry approach and the high incidence of pre-eclampsia in the high-risk women studied enabled us to find the statistically significant association between pan-African ancestry and early-onset pre-eclampsia. Individual genetic ancestry estimates are precise measurements of the components of ancestry and are advantageous compared to simpler approaches, such as principal components, which are mathematical constructs, expressed on relative scales and whose association with outcomes are not reproducible across different populations.

An additional strength of this study is the depth and quality of phenotyping. In contrast to the majority of genetic studies of pre-eclampsia and HDP which are reliant on electronic health records and ICD-10 codes [32], the diagnoses in this cohort were made by trained obstetricians using robust definitions from internationally accepted guidelines [3]. The availability of additional clinical variables also enabled inclusion of standard clinical risk factors to pre-eclampsia risk models, which previous genetic studies have not attempted [32].

Whilst this is the largest study of individual genetic ancestry estimates and pre-eclampsia risk to date in a multi-ancestry population, the sample size is modest. We did not perform an a priori sample size calculation, including all eligible women that were recruited between 2013 and 2021 in this study. Furthermore, the population was predominantly pregnant women with pre-existing (chronic) hypertension. Therefore, our findings need validation in an independent, multi-ethnic population that includes primarily normotensive women without known cardio-metabolic disease at entry to pregnancy. In addition, West African (Yoruban) ancestry was the most prevalent population African ancestry in the study cohort. Due to low prevalence of other genetically-computed African population ancestries, we were unable to determine whether the observed association with early-onset pre-eclampsia is specific to West African (specifically Yoruban) population ancestry, or whether we were simply underpowered to detect associations for other African ancestries. This study may be informative for risk associated with the most prevalent African ancestries present in contemporary UK society, but prioritisation of multi-ethnic cohorts in future genetic studies, will enable more definitive conclusions about the connection of ancestry, genetic variants and pre-eclampsia disease risk.

To our knowledge, only one other study has examined the association between genetic ancestry and the risk of pre-eclampsia. This was a study of 125 women with gestational hypertension or pre-eclampsia or Haemolysis, Elevated Liver Enzymes and Low Platelets (HELLP) syndrome or controls (n = 161) in Hispanic women living in the United States. Genetically-computed pan-African ancestry proportion was also associated with a higher risk for hypertensive disorders of pregnancy, and Native American ancestry proportion was associated with reduced risk in adjusted models [33]. Although a very different population to our study and timing of onset of pre-eclampsia was not reported, the consistency of finding an association between genetically-computed pan-African ancestry and hypertensive disorder of pregnancy risk adds further credibility to our findings. Specific African population ancestries were not investigated in this study.

It is notable that we only observed an association between genetically-computed ancestry and early-onset pre-eclampsia. Early-onset pre-eclampsia has the highest risk of recurrence in a future pregnancy [4], and stronger familial patterns of inheritance [34], consistent with higher likelihood of a genetic contribution to disease risk. It is also of interest that our data are consistent with the possibility that variants specific to West African ancestry populations may be associated with pre-eclampsia disease risk. There has been a growing understanding of APOL1 genotypes in West African populations and kidney and cardiovascular risk, and this interest has recently extended to pre-eclampsia [35]. APOL1 genotypes could be one mechanism by which West African ancestry specifically is associated with pre-eclampsia risk on a genetic pathway.

Whilst the optimal definition and computation of genetically-computed ancestry remains a subject of debate [17], [36], our findings agree with the critique of the use of self-identified ethnic groups as a reliable proxy for heritable or ‘biological’ risk. As well as discrepancy between self-defined ethnicity and genetically-computed ancestry as we have shown here, the categorical grouping of ethnicity is at odds with the biological reality of ancestry which is a complex, continuous variable. This view has been recently endorsed by the National Academies of Sciences, Engineering and Medicine (NASEM) and the American Journal of Human Genetics, which have both recommended self-reported ethnicity should not be used as a proxy for genetic ancestral groups and encourage the reporting and use of genetic ancestry in genomic studies [37]. This suggests that the use of ethnicity groupings in pregnancy risk prediction including pre-eclampsia should be reviewed [38].

## 5 Clinical perspective

Our data demonstrated a strong association between genetically-computed pan-African ancestry and early-onset pre-eclampsia that was independent of self-reported ethnic group. Further studies in large, multi-ethnic, well-phenotyped cohorts are required to verify the association presented here. Larger cohorts will allow exploration of prediction performance of genetic ancestry estimates compared to genome-wide polygenic risk scores and may allow finer mapping of population-specific causal genomic regions and variants. Untangling the causal pathways underlying the well-described epidemiology between maternal ethnicity and pre-eclampsia, definitively determining what maternal ethnicity is acting as a proxy for in this context, has the potential to greatly improve our understanding of pre-eclampsia and related disorders, risk prediction and clinical outcomes.

## Declaration of competing interest

The authors declare that they have no known competing financial interests or personal relationships that could have appeared to influence the work reported in this paper.
