# Supplementary material for: Association of genetic ancestry with pre-eclampsia in multi-ethnic cohorts of pregnant women
Source: Pregnancy Hypertens. 2024 Dec;38:None. doi: 10.1016/j.preghy.2024.101162 (PMC11870846; doi:10.1016/j.preghy.2024.101162)
Supplement: Supplementary Data 1 [file mmc1.docx]

**Supplementary methods**

*Genotyping*

Buffy coats were collected from venous EDTA samples following centrifugation (10 minutes at 2500xg at 4°C) and stored at -80°C. DNA was extracted, quantified, and normalised from buffy coat samples and genotyped using the Infinium® Global Screening Array by LGC Genomics (LGC Limited©). Genome-wide genotyping was performed in two batches. The first batch of samples (PEACHES cohort only) were genotyped on the Global Screening Array v1.0, and the second batch (PEACHES and MAVIS cohorts) were genotyped on the Global Screening Array v3.0. The Infinium® Global Screening Array contains approximately ~700,000 variants, designed to achieve a multi-ethnic genome-wide backbone optimised for imputation performance across populations.^31^

*Genotype quality control*

Sample and variant quality control filters were applied to the two batches of genotype data files separately to standards recommended for Genome-Wide Association Studies^32^ with some minor modifications. Samples were filtered for: genotype call rate ≥ 99%, sex discrepancies and duplicates. Autosomal Single Nucleotide Polymorphisms (SNPs) were filtered for Minor Allele Frequency (MAF) ≥ 5% and < 1% missingness across samples. Samples were not filtered for heterozygosity or Hardy-Weinberg equilibrium to prevent the removal of heterozygous variants that may be informative for ancestry calculation. Of approximately 300,000 SNPs that passed QC in both batches, 276,878 were present in the 1000 Genomes reference population genotype dataset and were selected to allow merging of the datasets prior to genetic ancestry estimation. All quality control steps were performed in PLINK 1.9 (www.cog-genomics.org/plink/1.9/).^33^

*Genetically-computed individual ancestry estimation*

Global genetic ancestry scores were calculated for each individual from genotype data using ADMIXTURE software. ADMIXTURE uses a maximum-likelihood version of the Pritchard-Stephens-Donnelly population genetics model.^34,35^ To allow estimation of genetic ancestry relative to reference populations, genotype data from the study cohorts were merged with genotype data from Phase 3 of the 1000 Genomes Project.^36^ N = 10,889 fully independent SNPs (r^2^ < 0.01) were used for genetic ancestry estimates.

Supervised estimation of global genetic ancestry was undertaken by specifying the population labels of the 1000 Genome project individuals as reference groups (Supplementary Table 2) in the ADMIXTURE model and setting the putative number of ancestral populations (K) as the number of population labels (K = 26). Following this, ADMIXTURE estimated ancestry proportions in non-reference individuals. Ten iterations of the model were performed with a random seed to assess the stability of ancestry estimates. Iteration results demonstrated stable ancestry estimates across linkage disequilibrium thresholds and are illustrated in Supplementary Figure 1. Using individual 1000 Genome populations (E.g. YRI – Yoruban), genetic ancestry estimates were summed into five superpopulation groups: African (AFR), Admixed American (AMR), East Asian (EAS), European (EUR), and South Asian (SAS) (Supplementary Table 2). The output per individual in the study cohort was percentage genetic ancestry/ies, by default summing to 100% (E.g. 50% African, 50% European). To aid interpretation, genetic ancestry estimate percentages were categorised into the following ‘equivalent grandparent(s)’ categories, with the notion that on average, an individual with genetic ancestry estimate percentage in the relevant category would have this number of grandparents from this ancestral group: < 5% - 0 grandparents, 5-24.9% - 1 grandparent, 25-49.9% - 2 grandparents, 50-74.5% - 3 grandparents, 75%+ - 4 grandparents. This is a theoretical construct to aid interpretation, as in practice individuals could inherit partial population ancestries from multiple grandparents.

1. **Supplemental Figures and Figure Legends**

**Supplementary Figure 1.** Assessment of stability of supervised ancestry estimates. *Data are plotted as aligned stacked assignment bar plots of individual supervised genetic ancestry estimates (% from each ancestral cluster with each cluster denoted by a different colour in the bar plot) computed using linkage disequilibrium threshold r^2^ < 0.01 (N = 10,889 SNPs). The study group are labelled according to 1000 genome project population codes (for code descriptors see Supplementary Table 2).* *All individuals are shown on a single row. Each row represents a single run of ADMIXTURE software (total runs = 10). The high consistency of clustering patterns and ancestral estimates across runs indicates stable algorithm performance.*

**
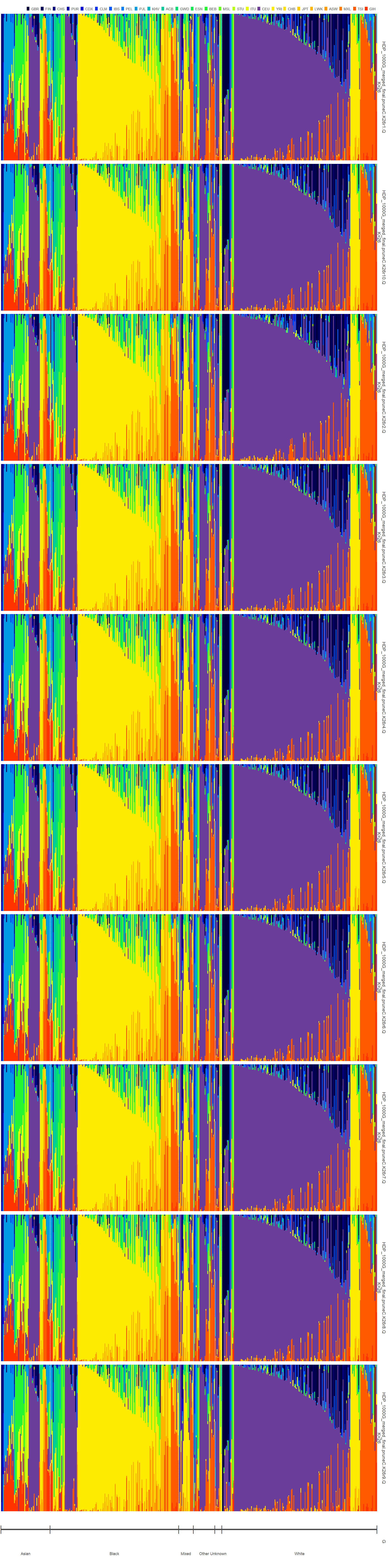
**

**Supplementary Figure 2.** Correlation between individual genetic ancestry A) superpopulation, B) population estimates in whole study group (n = 436).

1. B.

**Supplementary Figure 3.** Pie chart of 1000 genome population ancestry estimates across the whole study group (n = 436).

*Please see supplementary Table 2 for population label names.*


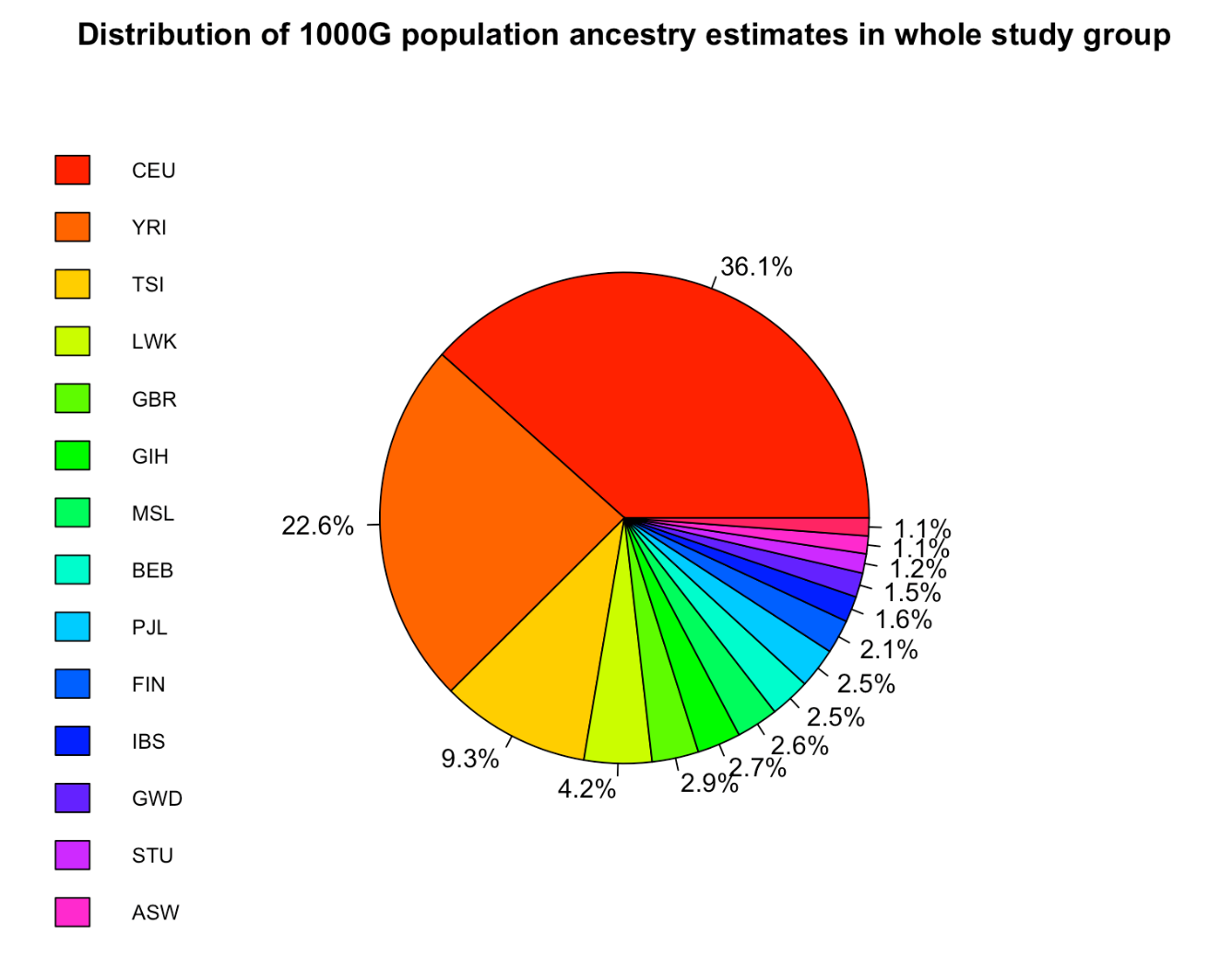

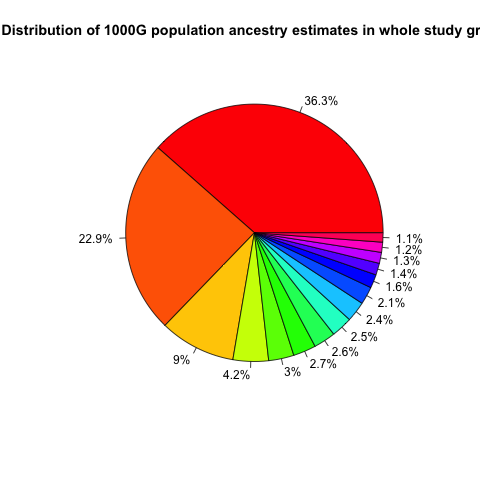


**Supplementary Figure 4.** Supervised genetically-estimated individual ancestry proportions, each population (1000 genomes reference populations) denoted by a three letter code (see Supplementary Table 2). Each individual is denoted by a single vertical stacked bar, with individual population ancestry percentage (on scale of 0-1) denoted by bar colouring. Individuals are grouped by self-reported ethnic group.


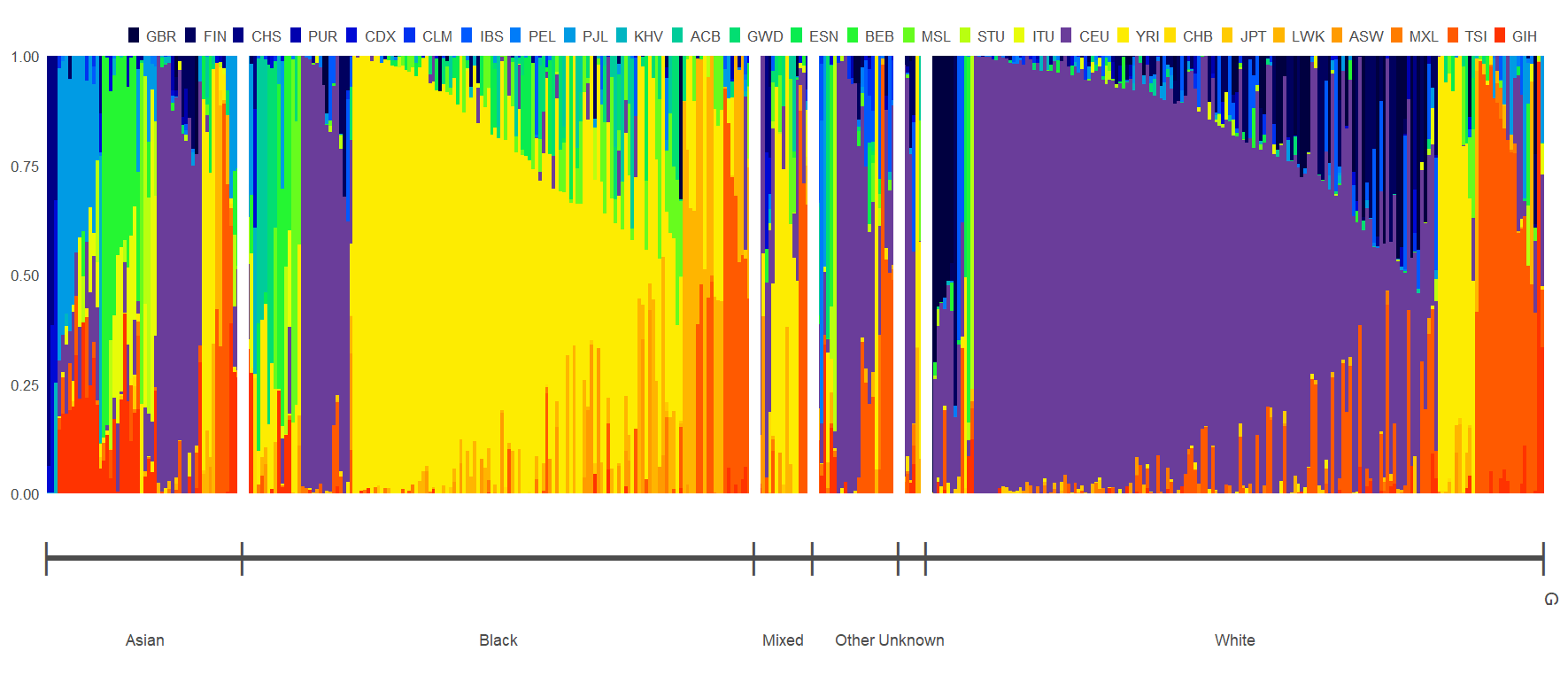


1. **Supplementary Tables**

**Supplementary table 1.** List of recorded ethnic categories in PEACHES and MAVIS cohort studies, and harmonised study categories. *ONS = Office of National Statistics, FMF = Fetal Medicine Foundation.*

| **PEACHES cohort ethnic categories** | **MAVIS cohort ethnic categories** | **Detailed study categories** | **Collapsed ONS study categories** | **FMF pre-eclampsia risk prediction categories** |
| --- | --- | --- | --- | --- |
| African, Sub-Saharan  African, North | African (Black/Black British)  Other Black background | African | Black | Black |
| Caribbean | Caribbean (Black/Black British) | Caribbean | Black | Black |
| Chinese  Other Far East | - | East Asian | Asian | East Asian |
| Bangladeshi  Indian  Pakistani | Bangladeshi (Asian/Asian British)  Indian (Asian/Asian British)  Pakistani (Asian/Asian British) | South Asian | Asian | South Asian |
| South East Asian | - | Other Asian | Asian | Other/Unknown |
| - | Other Asian | Other Asian | Asian | Other/Unknown |
| Mixed Caribbean-European  Mixed Other African or Caribbean  Mixed African-European  Mixed Asian European | Mixed White & Black Caribbean  Mixed White & Black African  Mixed White & Asian  Mixed other | Mixed | Mixed | Mixed |
| Other  Middle East | Other | Other | Other | Other/Unknown |
| European, British  European, East  European, Irish  European, North  European, South  European, West | British (White)  Other White  Irish (White) | European | White | White |
| Unclassified | Not disclosed  Missing | Unknown | Unknown | Other/Unknown |

**Supplementary Table 2.** Population and superpopulation codes and descriptions of 1000 Genome individuals.

| **Population Code** | **Population Description** | **Super Population Code** |
| --- | --- | --- |
| CHB | Han Chinese in Beijing, China | EAS: East Asian |
| JPT | Japanese in Tokyo, Japan | EAS |
| CHS | Southern Han Chinese | EAS |
| CDX | Chinese Dai in Xishuangbanna, China | EAS |
| KHV | Kinh in Ho Chi Minh City, Vietnam | EAS |
| CEU | Utah Residents (CEPH) with Northern and Western European Ancestry | EUR: European |
| TSI | Toscani in Italia | EUR |
| FIN | Finnish in Finland | EUR |
| GBR | British in England and Scotland | EUR |
| IBS | Iberian Population in Spain | EUR |
| YRI | Yoruba in Ibadan, Nigeria | AFR: African |
| LWK | Luhya in Webuye, Kenya | AFR |
| GWD | Gambian in Western Divisions in the Gambia | AFR |
| MSL | Mende in Sierra Leone | AFR |
| ESN | Esan in Nigeria | AFR |
| ASW | Americans of African Ancestry in SW USA | AFR |
| ACB | African Caribbeans in Barbados | AFR |
| MXL | Mexican Ancestry from Los Angeles USA | AMR: Admixed American |
| PUR | Puerto Ricans from Puerto Rico | AMR |
| CLM | Colombians from Medellin, Colombia | AMR |
| PEL | Peruvians from Lima, Peru | AMR |
| GIH | Gujarati Indian from Houston, Texas | SAS: South Asian |
| PJL | Punjabi from Lahore, Pakistan | SAS |
| BEB | Bengali from Bangladesh | SAS |
| STU | Sri Lankan Tamil from the UK | SAS |
| ITU | Indian Telugu from the UK | SAS |

**Supplementary Table 3.** Genetically-estimated African, European and South Asian ancestry percentage groups (0 to 4 equivalent grandparents (GP)) in whole study cohort (n = 436) stratified by self-reported ethnic category.

1. European ancestry

| **Self-reported ethnic category** | **Percentage (%) European ancestry group** | | | | |
| --- | --- | --- | --- | --- | --- |
|  | **<5% (0 GP)** | **5-24.9% (1 GP)** | **25-49.9% (2 GP)** | **50-74.9% (3 GP)** | **75% + (4 GP)** |
| White (n = 180) | 12 (6.7%) | 3 (1.7%) | 4 (2.2%) | 2 (1.1%) | 159 (88.3) |
| Black (n = 149) | 98 (65.8%) | 20 (13.4%) | 10 (6.7%) | 4 (2.7%) | 17 (11.4%) |
| Asian (n = 57) | 14 (24.6%) | 24 (42.1%) | 4 (7.0% | 2 (3.5%) | 13 (22.8%) |
| Mixed (n = 17) | 4 (23.5%) | 2 (11.8%) | 5 (29.4%) | 3 (17.6) | 3 (17.6%) |
| Other (n = 25) | 5 (20.0%) | 2 (8.0%) | 1 (4.0%) | 4 (16.0%) | 13 (52.0%) |
| Unknown (n = 8) | 3 (37.5%) | 1 (12.5%) | - | - | 4 (50.0%) |

1. African ancestry

| **Self-reported ethnic category** | **Percentage (%) African ancestry group** | | | | |
| --- | --- | --- | --- | --- | --- |
|  | **<5% (0 GP)** | **5-24.9% (1 GP)** | **25-49.9% (2 GP)** | **50-74.9% (3 GP)** | **75% + (4 GP)** |
| White (n = 180) | 163 (90.6%) | 3 (1.7%) | 1 (0.6%) | 1 (0.6%) | 12 (6.7%) |
| Black (n = 149) | 25 (16.8%) | 1 (0.7%) | 4 (2.7%) | 12 (8.1%) | 107 (71.8%) |
| Asian (n = 57) | 48 (84.2%) | 2 (3.5%) | - | 2 (3.5%) | 5 (8.8%) |
| Mixed (n = 17) | 3 (17.6%) | 3 (17.6%) | 3 (17.6%) | 2 (11.8%) | 6 (35.3%) |
| Other (n = 25) | 18 (72.0%) | 4 (16.0%) | 1 (4.0) | - | 2 (8.0%) |
| Unknown (n = 8) | 5 (62.5%) | - | - | - | 3 (37.5%) |

1. South Asian ancestry

| **Self-reported ethnic category** | **Percentage (%) South Asian superpopulation ancestry** | | | | |
| --- | --- | --- | --- | --- | --- |
|  | **<5% (0 GP)** | **5-24.9% (1 GP)** | **25-49.9% (2 GP)** | **50-74.9% (3 GP)** | **75% + (4 GP)** |
| White (n = 180) | 159 (88.3%) | 15 (8.3%) | 2 (1.1%) | 1 (0.6%) | 3 (1.7) |
| Black (n = 149) | 137 (91.9%) | 5 (3.4%) | - | - | 7 (4.7%) |
| Asian (n = 57) | 23 (40.4%) | 2 (3.5%) | 1 (1.8)% | 6 (10.5%) | 25 (43.9%) |
| Mixed (n = 17) | 15 (88.2%) | 2 (11.8%) | - | - | - |
| Other (n = 25) | 20 (80.0%) | 1 (4.0%) | - | 1 (4.0%) | 3 (12.0%) |
| Unknown (n = 8) | 7 (87.5%) | 1 (12.5%) | - | - | - |
